# Processing and Quality Control of Masks: A Review

**DOI:** 10.3390/polym14020291

**Published:** 2022-01-11

**Authors:** Sedigheh Farzaneh, Mohammadali Shirinbayan

**Affiliations:** 1 P4Tech, 23 Rue du 8 Mai 1945, 94470 Paris, France; sedigheh.farzaneh@gmail.com; 2 Arts et Metiers Institute of Technology, CNAM, PIMM, HESAM University, 75013 Paris, France

**Keywords:** face mask, medical devices, additive manufacturing, filtration performance

## Abstract

It is clear that viruses, especially COVID-19, can cause infection and injure the human body. These viruses can transfer in different ways, such as in air transfer, which face masks can prevent and reduce. Face masks can protect humans through their filtration function. They include different types and mechanisms of filtration whose performance depends on the texture of the fabric, the latter of which is strongly related to the manufacturing method. Thus, scientists should enrich the information on mask production and quality control by applying a wide variety of tests, such as leakage, dynamic respiratory resistance (DBR), etc. In addition, the primary manufacturing methods (meltblown, spunlaid, drylaid, wetlaid and airlaid) and new additive manufacturing (AM) methods (such as FDM) should be considered. These methods are covered in this study.

## 1. Introduction

Over the past decades, having a healthy body has been a critical need in which different facilities and personal protection devices were developed. These devices can protect people against micro-organisms and biological aerosols, including bacteria, viruses and fungi, which are recognized as a part of causing diseases. In addition, these days, a new virus called COVID-19 has been detected that has caused many deaths worldwide [1] and has been associated with other biological effects [2,3]. Regarding this disease, more demands for adopting personal protection equipment (PPE) is required. There is a wide variety of transmission ways of micro-organisms, such as airborne and direct/indirect contact, which is classified based on particle diameters. For example, airborne transmission is defined for particle diameters of ≤5 µm. This form of transmission spreads without contact and raises demands for facial protection such as face masks. This transmission can happen either between healthcare workers and patients, or in different indoor areas [4].

For this fact, excellent protection by face masks in the atmosphere against particles and aerosols leads emphasis on research and development in processing and quality control of face masks [3]. For instance, the type of polymer for fabrication plays an important role both in the final performance of face masks and the environmental risks [5,6,7,8]. Besides basic, industrialized fabrication manners of face masks such as meltblown, spunlaid, drylaid, wetlaid and airlaid technologies, cutting-edge processes of additive manufacturing (AM) processes are applied to meet demands, which are discussed in the following subsections.

For quality control, different experiments have been performed on filtration, leakage, dynamic breathing resistance (DBR) performance, wearing comfort, etc. [9]. Each test has its related terms that should be understood and explained. For example, for the infiltration performance test, the mechanism of inertial impaction, interception, diffusion and electrostatic attraction have impacts, which are presented in this review paper.

## 2. Types of Applied Materials in Face Mask Production 

Generally, in various investigations, it was explained that most starting materials for face mask fabrication include non-woven materials such as polypropylene, glass papers and woolen felt, which have been proven to have special characteristics such as high-temperature resistance in autoclaving while serving a stable structure and cost-effective final product [10,11]. Furthermore, disposable non-woven fabrics are another useful type of non-woven material that have gained attention due to the lower risk of contamination in comparison with other materials. In this regard, a comparison of some characteristics of this type with reusable materials was performed, as shown in Table 1. Reusable fabrics could also be sterilized for secondary applications [12].

However, generally speaking, usual applicable polymers in face mask production are polypropylene, polyethylene, polyesters, polyamides, polycarbonates, polyphenylene oxide and trifluorochloroethylene. Besides, some materials are applied together for better achievement of properties such as using polypropylene that is treated with dimethyl-dioctadecyl-ammonium bromide to improve bacterial attraction in order to import positive electrical charges [1].

### Characteristic of Non-Woven Fabrics

Based on previous explanations, most non-woven materials are disposable and single-use; however, the second group needs sterilization before reuse. Nevertheless, there are some advantages and limitations to the application of non-woven materials. The main characteristic of these materials is the low cost of the final product. Furthermore, their permeability to air and non-adherence to wounds makes them an excellent dressing material [13,14]. Nevertheless, the term “single-use” is a limitation regarding their low resistance and poor drape ability in consideration of disposable non-woven materials [15]. At this stage, non-woven materials consist of various characteristics, which are listed below, and affecting the mask structure:

**Fiber bonding.** Non-woven materials are usually fabricated by the addition of an external chemical binder. Mechanical bonding has a negligible effect on the absorbency of fibers since inherent characteristics are not involved in this type of bonding. Yet, mechanical bonding causes two changes in the entanglement of fibers. First, the entanglement could limit the natural ability of the whole structure to swell. Second, the structure may prevent collapse in presence of external pressure. Considering these changes, mechanical bonding influences the capillary absorption of fluid [15,16]. 

**Web assemblage.** The manner of fiber arrangement to form a structure has a significant influence on the web properties, including packing, capillary orientation, pore size, capillary dimensions, etc. The absorbency of non-woven fibers is considered to be affected by their arrangement as well. Localized rearrangement of fibers also fulfills web formation and increases the wicking abilities of fabric [15,16].

**Web finishing.** In the nonwoven method, the fibers are assembled into the final structure and bonded by chemical or physical means. The absorbency of the nonwoven compound increases by chemical finishing since it modifies the wetting performance of a fiber surface and, as a result, affects the capillary behavior. Mechanical softening treatments can affect web properties and absorbency characteristics since fiber crimp could have an influence on packing efficiency and the resulting structure [13,14].

**Fiber finishing.** Fiber finishing is used to improve fiber’s processing performance within the equipment utilized for the transformation of fibers into a web. Since the finishing is on the surface of the fibers, it can influence wetting and liquid wicking and can have a direct impact on absorbency. Other morphological features such as surface rugosity and core uniformity can, in some cases, affect absorbency. In addition, the performance requirements in the fabrication of non-woven materials involve an optimization of different properties: liquid interaction, fabric flexibility and air permeability, and tensile properties [16].

## 3. Classification of Face Masks

Here, different classifications of face masks based on the application, materials and methods of production are presented. However, Table 2 shows different types of face masks with respect to their categories and relative properties.

Due to the COVID-19 consequences and application of the SFMs in different departments, categories of these types are discussed more. According to the ASTM F2100-11 standard, SFMs are generally categorized into three main groups: Level 1 (low) barrier, Level 2 (medium) barrier, and Level 3 (high) barrier. Level 1 has the lowest barrier of protection, while Level 3 has the highest barrier of protection. There are different criteria that have been implemented into the classification of SFMs:

**Bacterial filtration efficiency (BFE)**: This criterion is designed for measuring bacterial filtration efficiency of SFMs using Staphylococcus aureus as the challenge organism. Staphylococcus aureus is based on its clinical relevance as a leading cause of nosocomial infections. A higher bacterial filtration efficiency percentage indicates a better protection level for the patient and healthcare professionals against transmission diseases from the source of the patient and healthcare professionals.

**Breathing resistance**: This is used to determine the resistance of airflow through the masks. The SFM is subjected to a controlled flow of air. A lower breathing resistance illustrates a better comfort level to the end-user. The following sections will provide more information about this test.

**Quality evaluation**: This controls the quality evaluation to avoid transmission diseases, and the critical requirements are performed before the marketing of SFMs. For example, one of the important parts of the quality evaluation is the investigation of toxicity and biocompatibility of the masks. Sipahi et al. [23] studied the biocompatibility of eight marketed masks with different brands through their cytotoxicity and inflammation-inducing capacity. They showed that widely used disposable medical masks induced a surprisingly high rate of cytotoxicity and inflammation. In addition, they showed that evaluation of inflammation with cytotoxicity can be used to study the biocompatibility of medical devices such as with surgical masks.

Based on the mentioned classifications, there are three main levels of protection for SFMs that are indicated in Table 3.

## 4. Primary Techniques of Processing

**Figure 1:**
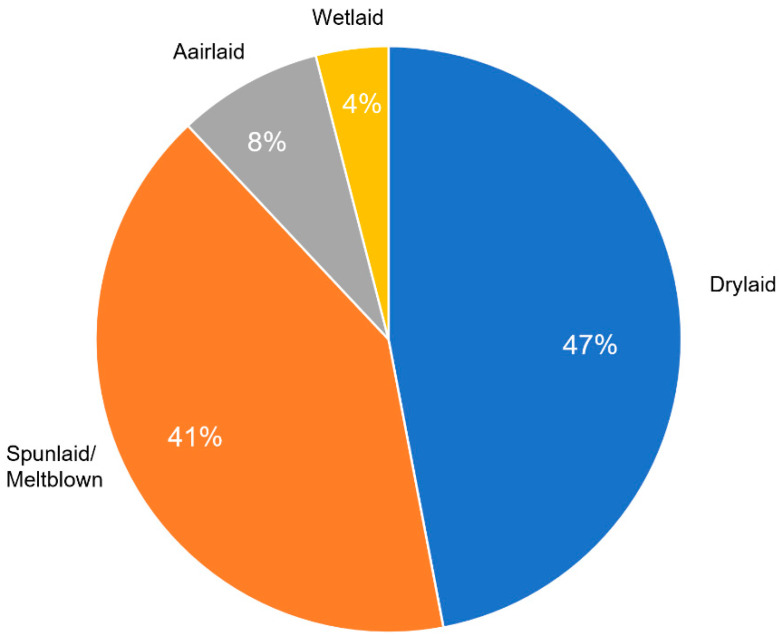
Repartition of worldwide production of nonwoven materials according to technologies [25].

During the past several years, different technologies have been implemented in the fabrication of non-woven fabrics [24,25,26]. As explained, the manufacturing of these materials are divided into two main steps: preparation of fibers in the web and bonding of fiber in the web. Presumably, there is related technology to the formation of a web that will be explained in this section. Repartition of worldwide production according to the technologies is shown in Figure 1. 

### 4.1. Meltblown Process and Spunlaid Technology

**Figure 2:**
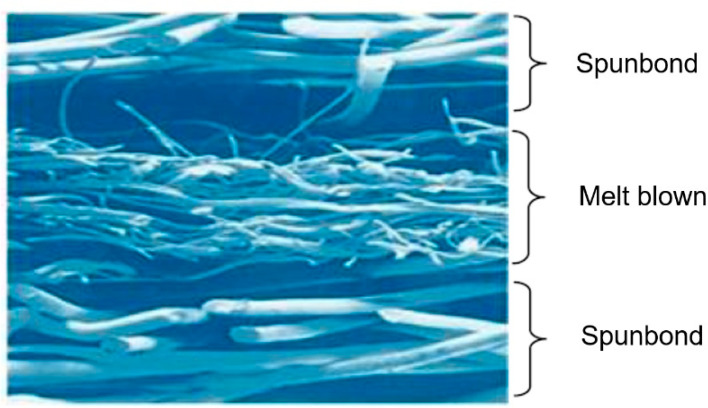
Typical microscopic image of a web representing the large fibers as spunbond and small fibers as meltblown [33].

The development of microfiber was first applied using a spray gun as a process to improve textile structures [27]. Following the expansion of microfibers, the technology was patented as a meltblown process [28,29]. In this process, numerous thermoplastic polymers such as polypropylene (PP), polystyrene, polyesters, polyurethane, nylon, polyethylene low and high density (LLDPE, LDPE, HDPE), and polycarbonate (PC) are used, of which the most popular polymer is polypropylene. Due to its low-melt viscosity, there is a possibility of passage through the micron-size holes. As an example, almost all meltblown webs are layered between two spunbond fabrics as shown in Figure 2 [30,31,32].

### 4.2. Meltblown Process

**Figure 3:**
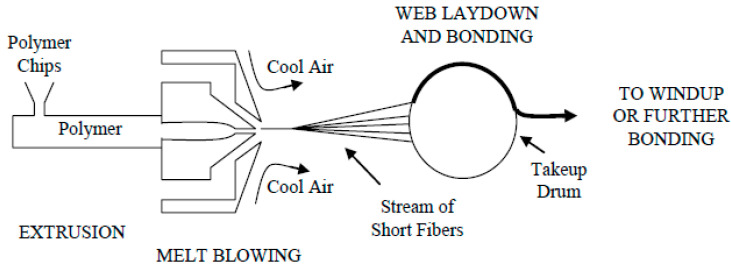
Schematic of the melt blowing process [35].

This process was first presented in the early 1950s by the United States Naval Research Laboratories and was applied to thermoplastics to produce microfibers of less than ten microns diameter [34]. In this process, there are four different factors: die assembly, the extruder, metering pump, and winding. The polymer resin is heated to melting point by feeding into the extruder, it then passes through the metering pump, turning into a homogenized polymer that feeds into the die assembly. As soon as the formation of a self-bonded web is performed, the microfibers are collected on a drum (Figure 3).

The definition and characteristics of applied polymers are not usually available, and hence researchers tried to publish the related works in order to perform the correlation of different parameters. However, it comprises a number of parameters, including machine, process, and materials. The interaction of these parameters is an important issue in the process, and the most important parameters are summarized in Table 4 [35].

**Figure 4:**
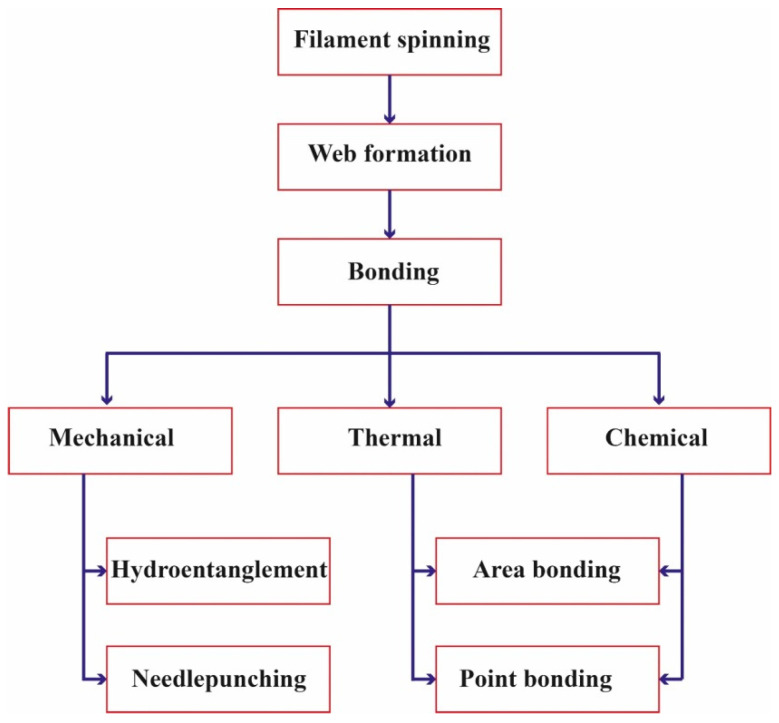
Sequences in spunlaid process [36].

**Spunlaid technology.** This technology, also called spunbond, is a machinery system adopted with polymer extrusion that manufactures fiber structures from molten filaments. These systems were presented commercially for the first time by DuPont and Rhone-Poulenc in the US and France in the mid-1960s, respectively [34]. This technique provides the chance for mass, cost-effective nonwoven products. There are different steps in this process that are illustrated in Figure 4.

**Figure 5:**
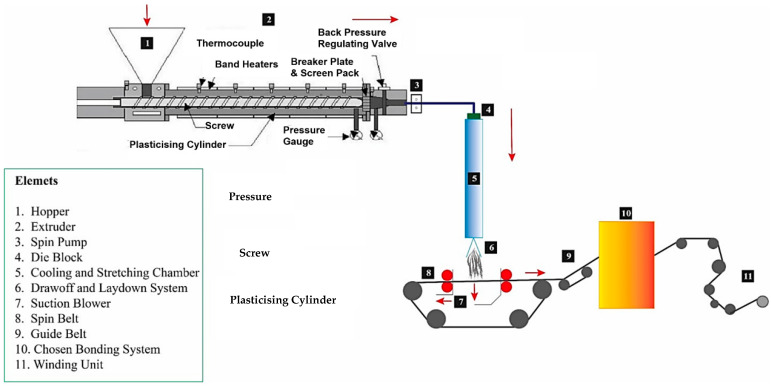
Schematic spunlaid process [36].

As can be seen in Figure 5, the spunlaid process contains different parts and elements for production, including extruder, filter, metering pump, spinning block, quenching, drawing, web forming, bonding and winding.

### 4.3. Drylaid Technology

This technology was first designed for textile industries, of which the common applicable materials comprise different staple fibers such as polyester, polypropylene, and cotton. Normally, the chosen fibers are those capable of reaching the web properties. Drylaid webs processing generally consists of four steps: (i) staple fiber preparation, (ii) opening, cleaning, mixing, and blending, (iii) carding, and (iv) web laying [34]. Although it is necessary to cut the produced fibers into staple fibers, the fiber preparation process is affected by the manufacturing methods. As these procedures are successive, the opening, cleaning, and mixing should be without defects in the products in order not to apply negative effects on the final product. The carding step is then performed by a machine named “card”. This step is conveyed by passing the entangled fibers between the closely spaced cloth surfaces [37].

### 4.4. Wetlaid and Airlaid Technology

**Wetlaid technologies.** These technologies come directly from paper-making technologies that are designed to manipulate fibers suspended in fluid, defined as “wetlaid". They were primarily introduced in the 1930s by Dexter and was purchased by Ahlstrom in 2000, who is a leader in wetlaid products [34]. The green material comprises cellulosic fibers as wood pulp and a wide variety of other fibers. These fibers have a short length in the size range of 2 to 20 mm. The process consists of dispersion to be as homogeneous as possible, the blend of fibers in water to flow the fiber solution into a forming wire, and the extraction water through the forming wire to lay fibers into a web form. Regarding the size and fineness of the fibers, the webs will look extremely uniform and sometimes similar to paper.

**Airlaid technologies.** The arrival of this technique dates to the 1960s, with Karl Krøyer in Denmark, and was subsequently sold to the M&J Fibertech company in the 1980s. This technology applies the same type of raw materials as wetlaid and particularly short firers as wood fibers. This process comprises obtaining a homogenous suspension of fibers in the air and then filtering this suspension through a forming wire. Fibers held by the wire will form the web. As for the wetlaid, the webs will look exceptionally uniform [34].

## 5. Additive Manufacturing (AM) of Face Masks 

**Figure 6:**
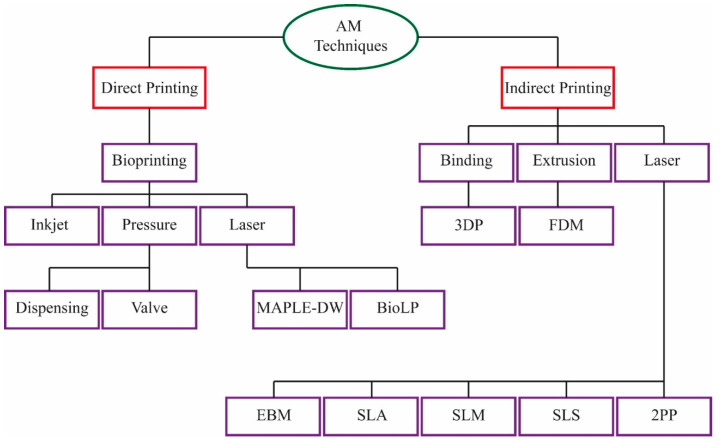
A review of additive manufacturing (AM) technologies and related subsessions [38].

Three-dimensional (3D) printing or additive manufacturing (AM) technologies (Figure 6) are known as the fourth industrial revolution in our scientific world, which was first presented in 1986 by Charles Hull through a manner of so-called stereolithography (SLA). This technology is expanding because of the wide variety of advantages such as minimum demands for postprocessing, less unusable wastes materials, and widely used applications, especially in polymers and face masks production [38,39]. AM technologies are replacing other technologies to become an accepted generic term for layer technology. Everyone is able to operate a 3D printing machine, even at home or inf an office, to print a 3D object [40]. Currently, AM machines play an important role in medical devices and biomaterial fabrication [41]. 

In polymeric materials, the critical limitation is the contaminations against viruses or bacteria in processing [41,42]. Currently, polylactic acid (PLA) is a common polymer in AM technologies produced from renewable sources [43].

**Figure 7:**
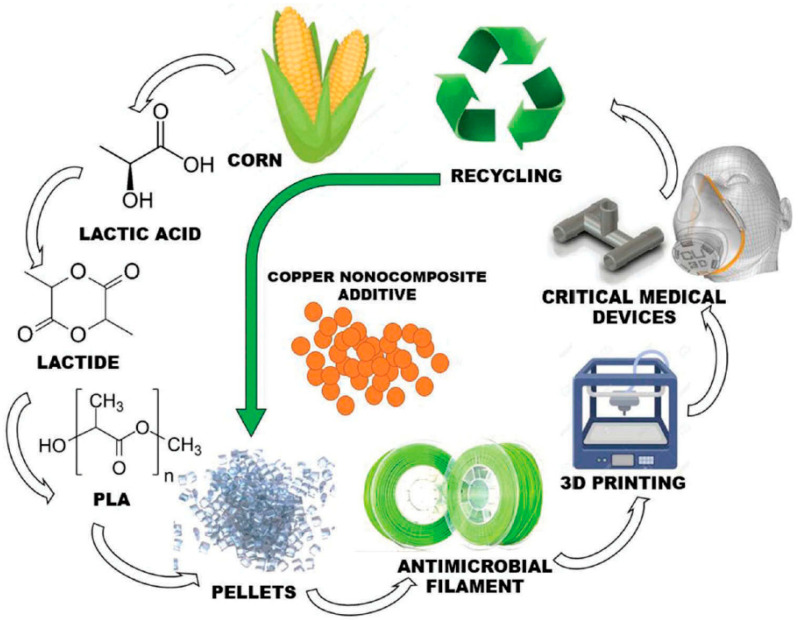
A representation of the manufacturing process for an antimicrobial polymer [44].

Apparently, there is a problem of sterilizing of the 3D-printed parts due to the porous structure in the range of 6–8 µm [44], which could be a weak point of using these structures in medical objects. However, applying the specific settings in the extrusion of layers to the antimicrobial materials can reduce dimensions to around 0.0002 µm, which is smaller than the size of viruses, such as in COVID-19 [44,45]. For instance, the 3D-printing and industrial production of PLA is presented in Figure 7.

**Figure 8:**
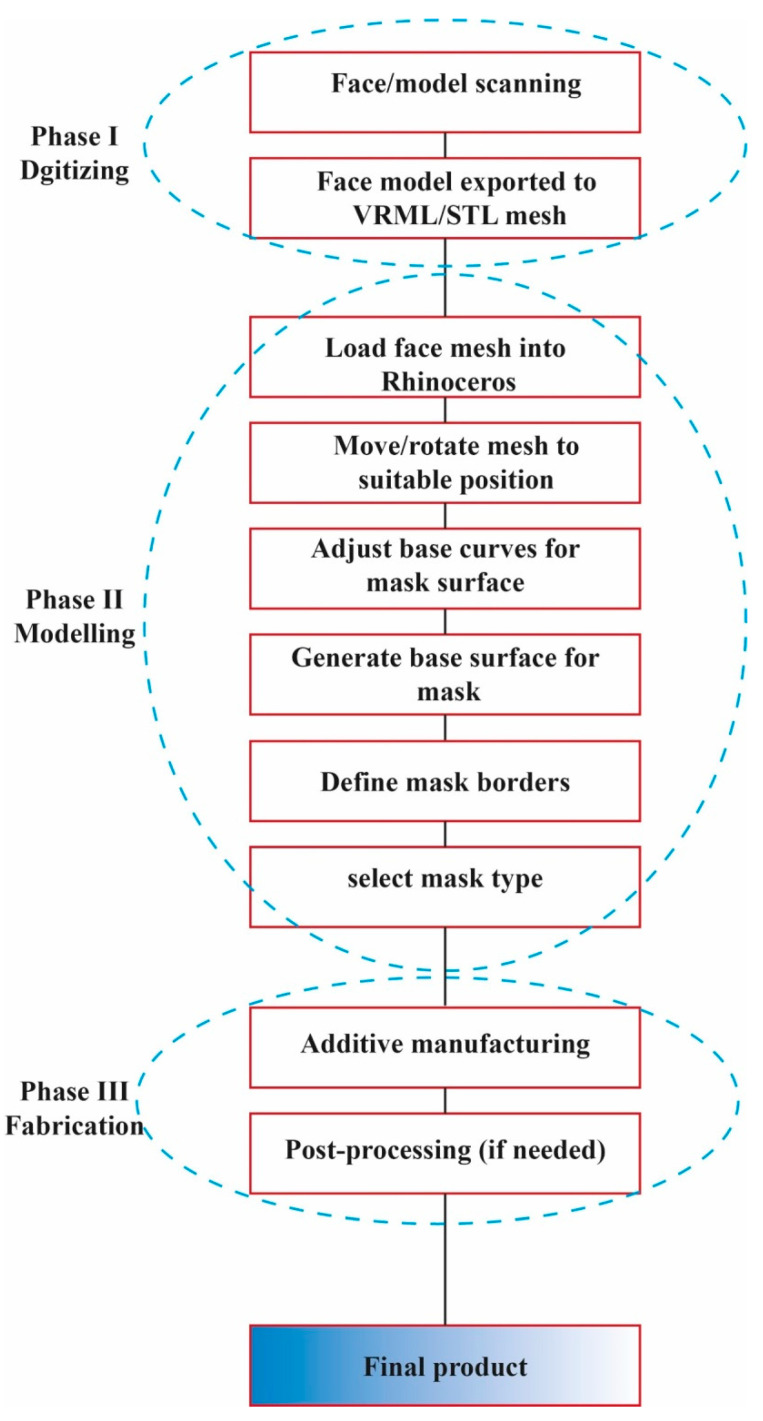
Methodology in additive manufacturing of a face mask [51].

Researchers are of the opinion that AM techniques could be used in the fabrication of medical devices to provide rapid production of final products such as ventilators, connectors, face masks, etc. [46]. As mentioned before, the importance of face masks for patients and health care workers is enticing because they categorize it as a critical medical device, especially against coronaviruses. There exists a limitation in full protection against viruses or bacteria due to the gap between the surfaces of the face masks and face (i.e., leakage) [47]. Despite the different efforts during the COVID-19 pandemic in the fabrication of medical devices [48,49,50], most researchers tried to propose the application of AM machines in the production of face masks in an efficient time. In Figure 8, general methodology workflow for face mask production is illustrated, which involves three main phases including: “Phase I”, “Phase II” and “Phase III” that are digitizing, modeling and fabrication, respectively.

**Figure 9:**
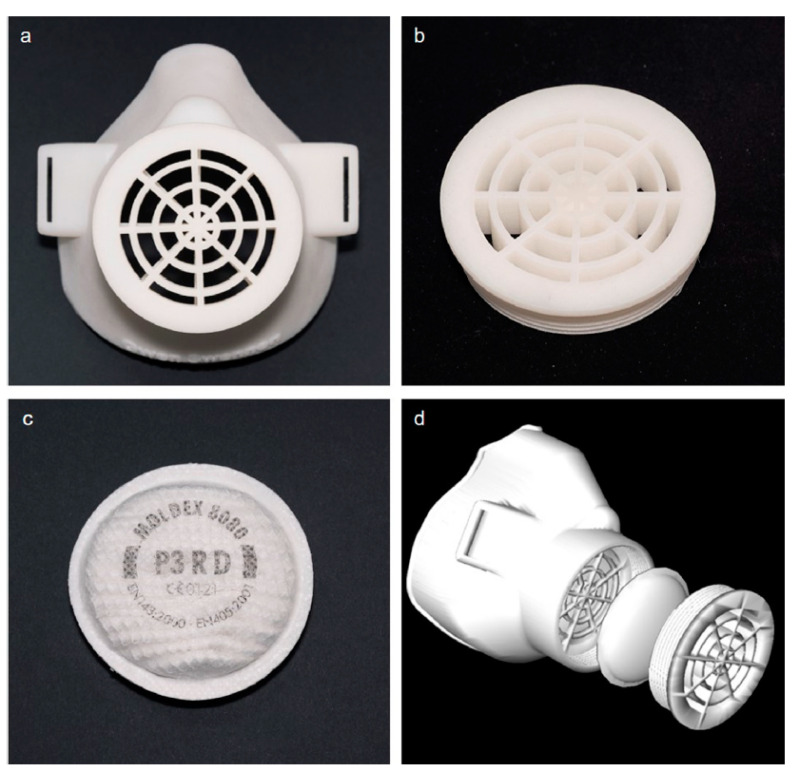
Typical representation of the 3D-printed face mask: (**a**) reusable 3D printed face mask, (**b**) filter membrane support, (**c**) polypropylene (PP) nonwoven meltblown particle filter, (**d**) 3D image of the prototype [52].

Swennen et al. [52] proposed a custom-made 3D printed face mask as a replacement against the lack of FFP2/3 SFMs. As shown in Figure 9, reusable polyamide 11 (PA11) was supplied for the 3D-printed SFM, and polypropylene (PP) non-woven meltblown particles were implemented for the filter membrane. They found that the 3D-printed SFM in combination with the FFP2/3 filter membranes could be an alternative; however, they require to show more validation of the proposed method.

**Figure 10:**
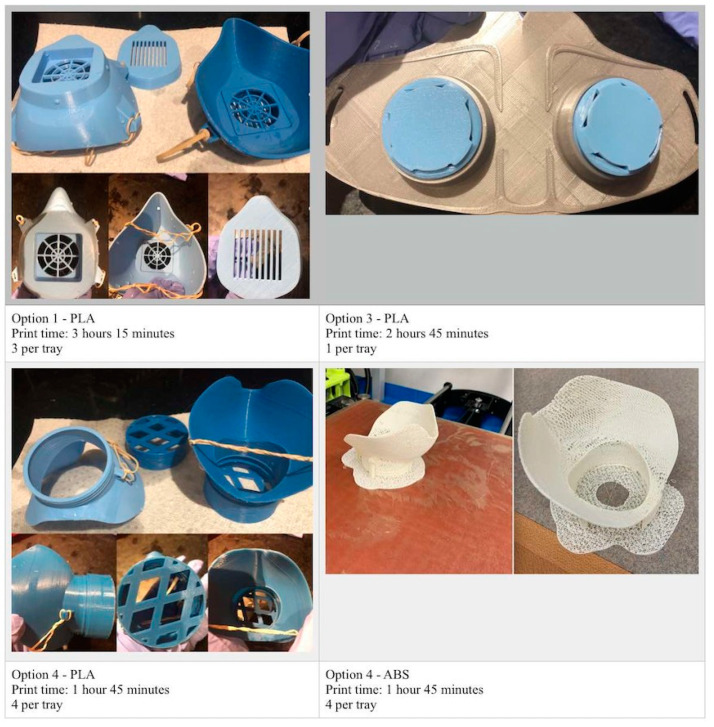
Typical image of fabricated masks using PLA and ABS [53].

Consequently, Provenzano et al. [53] worked on the fabrication of reusable 3D-printed N95 face masks in different conditions by using many 3D-printing machines with PLA and ABS. Based on the outcomes, they found that PLA has better quality in comparison with ABS (Figure 10). 

Finally, AM technology in the fabrication of face masks requires more developments in order to obtain high-quality products. However, recent efforts are appreciable to be applied in the medical industry for the appropriate applications.

## 6. Standards in Quality Controls of Face Masks 

Currently, due to this unrecognized virus (COVID-19), companies have produced a vast variety of masks; furthermore, minding the standards are essential. For this, the required information about the most important standards should be covered. The standard of “EN 14683:2019+AC:2019” is attributed to medical face masks (i.e., requirements and test methods), with the scope of construction, design, performance requirements and test methods for medical face masks that aim to decline the transition of ineffective agents from staff to patients. Table 5 presents the needed terms of SFMs for acceptable performance.

## 7. Filtration Performance (FP) Tests

**Figure 11:**
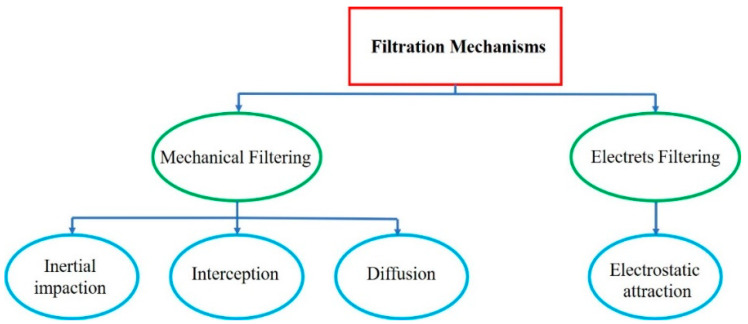
Different mechanisms in the filtration process [1].

The first step to perform quality control of a face mask is a filtration test, which plays an important role in mask quality evaluation. Different researchers consumed time for investigating this area, which introduced the mechanism of filtration in masks and respirators. In this protocol, four mechanisms work together: inertial impaction, interception, diffusion and electrostatic attraction. They are presented in Figure 11 [1,54]. 

**Figure 12:**
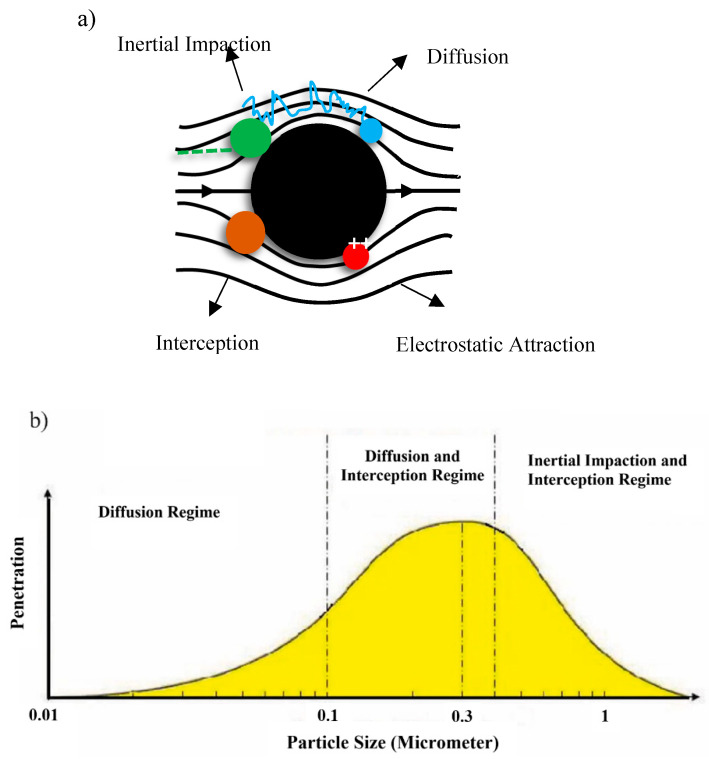
(**a**) Different collision of particles based on the four main filtering mechanisms. (**b**) The relationship between particle size distribution and type of filtering mechanisms [54,55].

The activation of each mechanism depends on particle size, face velocity and density in the airflow atmosphere. Figure 12 shows the relation of particle size and mechanisms of activation.

The mechanism of inertial impaction occurs when the size of the particle is more than 1 µm, which causes an increase of inertia in each particle, altering the direction of the particle in the atmosphere. The interception mechanism takes place when the particle size lowers to around 0.6 µm, which is not dependent on the face velocity of the particle, and no deviation is observed during the progress in comparison with former mechanisms. Besides, the most productive mechanism in the filtering of the particles is diffusion progress that accounts for particle sizes of less than 0.2 µm and in low velocity, based on the Brownian motion of particles. This motion increases the probability of particle accidents with fibers, and reduced velocity broadens the holding time of particles that consequently improves the probability of particle accidents and efficiency of filtering. Finally, the last mechanism is an electrostatic attraction that occurs by charging either the media or the particles, which is in addition to the mechanical mechanisms employed in NIOSH (National Institute for Occupational Safety and Health) accepted filters. In this mechanism, velocity has a negative impact on efficiency [55].

Filtration efficiency is defined as the capability and capacity of reserving viruses and particles in the atmosphere [6] and is related to different factors such as thermal rebound, face velocity, airflow rate, humidity and particle charge states, which are briefly described in Table 6 [56]. 

**Figure 13:**
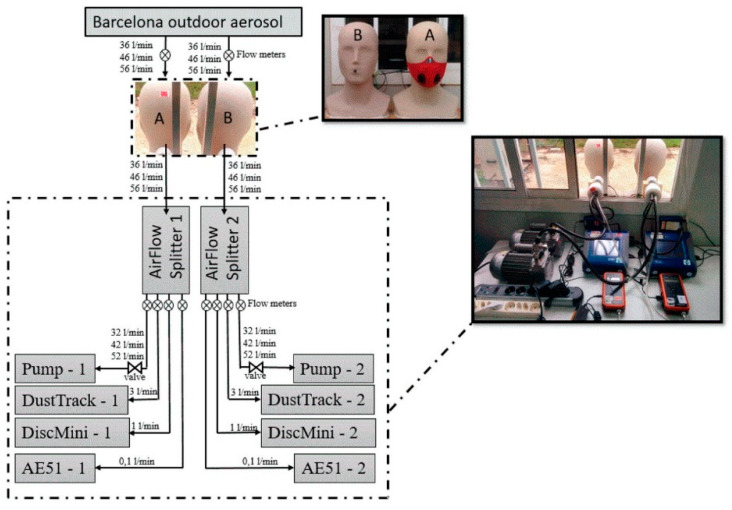
Set-up illustrations for measuring performance [58].

Besides these kinds of evaluation, Pacitto et al. [58] researched exposure evaluation of nine different face masks based on price (1–44 Euros) in the reduction of exposure to particle mass concentration (PM2.5), particle number concentration (PNC), lung deposition surface area (LDSA) and black carbon concentration (BC), with breathing rates of 32, 42 and 52 l.min^−1^. The test set-up is illustrated in Figure 13. Dummy heads were used as adult human heads in special dimensions, and they were occupied with different masks and different additional equipment such as airflow splitters, pumps, dust track, etc. The dummy heads were placed outdoors at a height of 1.60 m, and the mouth of each head employed an anti-electrostatic inlet tube and splitter separating airflow in 4 channels. It was reported that the effectiveness is directly related to the PM2.5 concentration.

## 8. Leakage Test

Besides using a filtration test, the leakage problem is another contributing factor that should be considered [59]. When the leakage happened, filter penetration disappeared, causing the consideration of this term to be studied as a part of the quality controlling of masks. For instance, Guha et al. [60] conducted a study to understand the contribution of leakage of aerosols through the gaps in SFMs and surgical respirators. They searched into the leakage of charge-neutralized, polydisperse, dried sodium-chloride aerosols in different personal protective equipment (PPE), with altering breathing rates, aerosol particle sizes and gap sizes. The ration of aerosols concentration between the input and output of SFMs is defined as intrinsic penetration without gaps, or total inward leakage (TIL), with consideration of gaps based on percentage. As mentioned, the protection is related to the intrinsic penetration and amount of leakage in the site. Generally, the summation of the two terms gives total inward leakage (TIL), and the penetration was separated from TIL from studying the effect of particle size on leakage. Thus, the leakage is noted as:

Leakage (size)%=TIL(size)-penetration(size)

**Figure 14:**
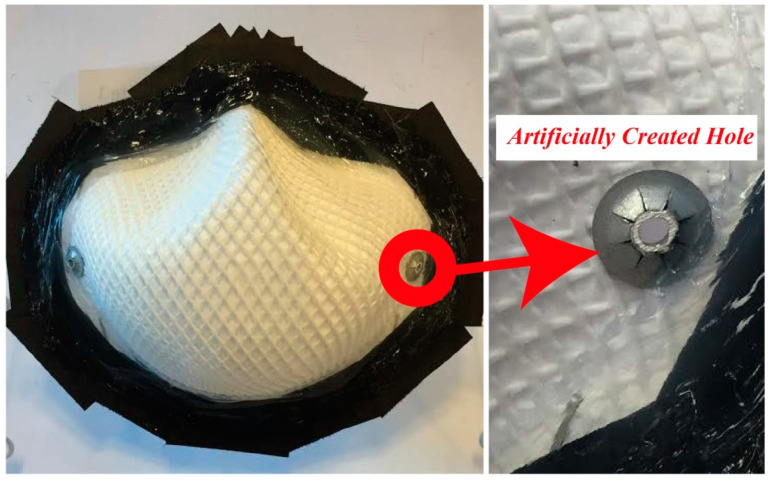
The holes are created to evaluate leakage performance of the mask [60].

For the experimental part, an artificial hole on the mask was created to perform the leakage test (Figure 14).

Finally, they announced that aerosol leakage is not related to size, especially above 100 nm in used masks. In addition, more TLI normally does not attribute to higher risk and is considered in parallel with the breathing flow rate [60]. 

**Figure 15:**
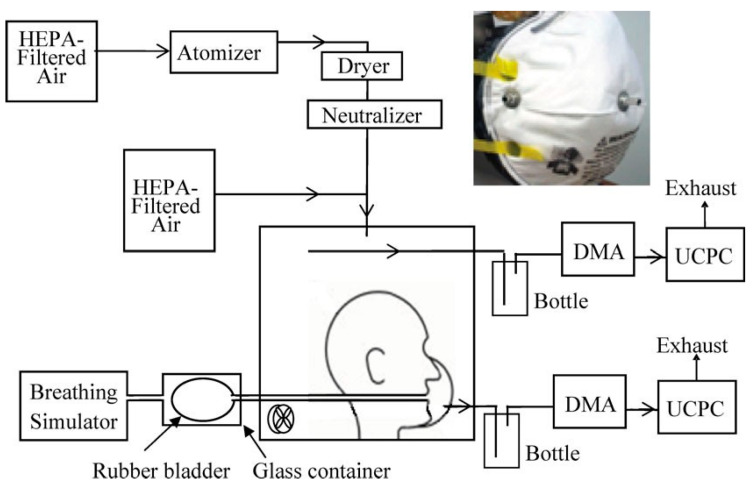
Experimental set-up for testing and evaluation of submicron-sized bioaerosols leakage [61].

For instance, Rengasamy et al. [61] researched the evaluation of filter penetration and face seal leakage to TIL with submicron-size bioaerosols (NaCl). In this study, different artificially created holes were placed into two N95 FFR models, using SFM models applied to a manikin that breathed minute volumes of 8 and 40 L. Figure 15 illustrates the set up of this research that two modes were investigated: (a) no artificial leaks and (b) with some artificial leaks induced through the needle. In addition, for better understanding of the research, the breathing simulator serves various changing terms, such as tidal volume and breathing rate.

Finally, the results showed that N95 FFRs outweigh the two SFMs in terms of filtration efficiency and good fitting characteristics.

## 9. Dynamic Breathing Resistance (DBR) Test

**Figure 16:**
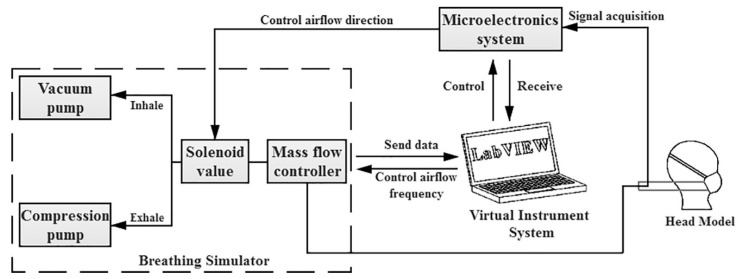
A designed experimental set up for measuring dynamic breathing resistance (DBR) [55].

Yao et al. [62] designed an experimental set-up including breathing simulator, mass flow controller, virtual instrument system, microelectronic system and head model (Figure 16). In addition, the role of each part is presented in Table 7.

Moreover, six indices were proposed to evaluate the dynamic performance of face masks in the breathing process, which is presented in Table 8.

Based on this research frame, twelve types of facemasks with various varieties, such as shape, respiratory valve, and basic materials, have been tested in which the results show that there are noteworthy differences between the indices in each type of applied mask. It was proven that the maximum breathing resistance of the dynamic measurement in comparison with the breathing resistance of the static measurement revealed a linear relationship. In addition, DBR provides an altered rate of breathing resistance [62]. 

## 10. Conclusions

The main purpose of this review was to present different techniques in quality control and processing of face masks. These days, due to the COVID-19 consequences, public attention is drawn to face mask application for reducing death and infection. For this, productive information about face masks in terms of starting materials, primary and advance processing, mechanisms of filtration and related required application tests were considered.

Face masks made of different polymers such as polypropylene, glass papers, woolen felt, polyethylene, polyesters, polyamides, polycarbonates, and polyphenylene oxide have the own properties, and they need more detailed evaluation. 

The families of face masks include basic cloth face masks, surgical face masks (SFMs), N95 respirator, P100 respirator/gas mask, self-contained breathing apparatus (SCBA), full face respirator and full-length face shield. Each type of face mask has special advantages concerning application. For instance, basic cloth face masks are easily fabricated materials (e.g., can be produced from a T-shirt, etc.) at low cost but lack efficient infiltration. Surgical face masks (SFMs) and N95 respirators show almost similar efficiency infiltration. For a P100 respirator, it was reported that the efficiency of filtering is 99.97% and presents less leakage, which is better than SFMs and N95.

For face mask fabrication, there are different methods such as airlaid, wetlaid, spunlaid/meltblown and drylaid, and each one shows specific properties. A new generation of fabrication methods called additive manufacturing (AM) is also applied for face mask production, which is expanding. However, AM techniques need more development to obtain high-quality products in terms of mechanical and physical properties. 

After face mask production, quality control is the final step before marketing. Generally, the tests of filtration performance (FP), leakage, and static/dynamic breathing resistance (DBR) are passed to inspect the efficiency of face masks. Different set ups for validation of face masks were presented and reviewed. 

In future studies, it is recommended to study the recycling of used face masks and mechanical properties of AM machined ones for enriching more information and improving the quality of face masks. Also created steam during the respiration cycle can provide the environment with high humidity which leads to the accelerated mechanism of penetration and faster spread of microorganisms to the inner parts of the mask. Regarding, production of masks to deal with this phenomenon, it seems necessary, especially in masks such as Surgical face masks (SFMs), Basic Cloth face masks and N95 respirators.

## Figures and Tables

**Table 1:** Comparison between disposable and reusable textiles characteristics [11].

Characteristic	Disposable Non-Woven	REUSABLE	
		Traditional Textile	Micro-Porous Textile
Mechanical behavior	1	2	3
Resistance to bacterial penetration	3	1	2
Resistance to liquid penetration	3	1	2
Flexibility	3	1	2
Remark: 1–3 represent poor to best criteria of properties.			

**Table 2:** Introduction to types of face masks [1,17,18,19,20,21,22].

Types	Pros and Cons	Appearance
Basic Cloth face masks	Easily fabrication, cost-effective and simplest type of face mask. The starting materials could be clothes sweatshirts, T-shirts, etc. However, not much applicability for aerosols with diameters of 20–1000 nm compared to the other types.	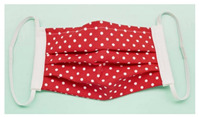
Surgical face masks (SFMs)	This type serves the wearer for protection against fluid stream and bacteria capturing. It has three layers, with a role of filtering media, moisture absorbance, and water repelling. The effectiveness of this type is similar to the N95 respirator. However, they are not capable of reducing the emission of small-size droplets.	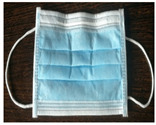
N95 respirator	Known as electrets filters in the group of filtering facepiece respirators (FFR), with surgical and standard sorts, they filter particles with diameters of 0.3 µm with 95% efficiency. It has a ventilator fan and four layers of materials of non-woven polypropylene for outer/inner layers and modacrylic, non-woven polypropylene metlblown for middle ones. However, N95 respirators are not applicable for sufficient protection against aerosols with diameters of less than 300 nm.	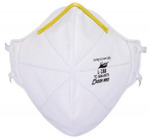
P100 respirator/gas mask	This is another type of filtering facepiece respirator (FFR), with a particle-filtering efficiency and penetration of 99.97% and 0.03%, respectively. In addition, this type is better than N95 respirators in terms of less leakage and keeping a standard form in changing temperature and humidity.	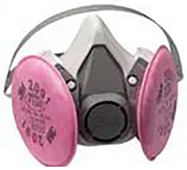
Self-contained breathing apparatus (SCBA)	This type of mask is equipped with an air supply that is normally applied for firefighting protection that resists forms of airborne contamination. However, it limits the mobility of the user and restricts workplace moments.	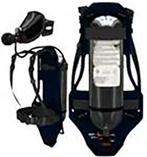
Full face respirator	This is made from rigid plastic materials with transparent parts for observation, which are fabricated for the aim of breathing trouble treatment. There are different types with respect to the size and shapes: air-purifying respirators (APR) and atmosphere-supplying respirators (ASR). Face supplies for holding the masks are made of adaptable elastomeric materials to well cover the face. Another element is straps that hold the mask body on the user head for the aim of leakage prevention. However, based on wearer behavior, these elements, especially the straps, can be broken.	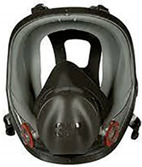
Full-length face shield	This kind of mask contains elastic headbands to cover the head and a transparent rigid polymeric (polycarbonate) full-length face shield. This could protect the user from liquid infected splashes in sneezing.	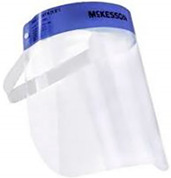

**Table 3:** ASTM F2100-11 levels of protection in SFMs.

Level of Protection	Characteristic of Each Level
Level 1 (Low barrier)	- Minimum BFE protection - Used for general procedures and respiratory etiquette - Designed to resist splash or spray at venous pressure
Level 2 (Moderate barrier)	- High BFE protection - More breathable than high barrier masks - Designed to resist a splash or spray at arterial pressure
Level 3 (High barrier)	- High BFE protection - Highest fluid resistance - Designed to resist a splash or spray during tasks such as orthopedic surgery

**Table 4:** Definition of machine, process, and material parameters [31,34,35,36,37].

Machine Parameters	Process	Material
Air velocity Air pressure Air temperature Die temperature Air flow rate Melt flow index	Polymer temperature Air temperature Die temperature Die hole size Die set-back Web collection type	Polymer forms (granules, chips) Polymer type Polymer degradation Polymer additives Melt viscosity -

**Table 5:** Demands for the excepted performance of medical face masks (EN 14683:2019+AC:2019).

Evaluation	Type I ^a^	Type II ^a^	Type IIR ^b^
Bacterial filtration efficiency (BEF), %	≥95	≥98	≥98
Differential pressure (Pa·cm^−2^)	<40	<40	<60
Splash resistance pressure (kPa)	NR *	NR	≥16.0
Microbial cleanliness (cfu. g^−1^)	≤30	≤30	≤30

* not required. ^a^ Classified as bacterial filtration efficiency, which is “type I” and should only be used for patients and other persons to limit infections spreads. ^b^ This type is divided with respect to splash resistance “R”.

**Table 6:** Test parameters and details of related roles in filtration efficiency [56,57].

Factors	Remarks
Thermal Rebound	Definition: Based on critical velocity and kinetic energy, which depends on particle diameter, yield pressure, particle density, etc.
	*Effects:* Negatively affect filtration efficiency in nanoscale particles, which depends on high temperature behavior of nanoparticles that is difficult to define the exact critical diameter of the boundary condition.
Face Velocity	Definition: Has an impact on diffusion, interception and electrostatic attraction of the fibrous filtration, which contributes to particle shape and velocity range.
	*Effects:* Generally, in high velocities (e.g., 20 cm.s^−1^), it causes an outweighing interception mechanism to become a diffusion mechanism, which reduces the filtration efficiency.
Airflow Rate	Definition: Used for filtration efficiency evaluation of respiratory and fibrous filtration.
	*Effects:* This factor directly increases the penetration of the particles by increasing airflow rate. The suggestion for the test is 85 and 350 l.min^−1^ for similarity with real situation.
Relative Humidity (RH)	Definition: In large scale particles, elevation in capillary force, which consequently improves the adherence of particles to the fibers in charged filters, takes a part with ions and electrons.
	*Effects:* Depending on the filtration mechanism, it has negative and positive impacts on the filtration process, which, in mechanical and electrets filtration, shows an increase and decrease in the process, respectively. Generally, it was reported that the type of effect is completely related to the fabrication of the masks and filters.
Particle Charge States	Definition: This considers charged/uncharged particles with mechanical and electrets filtration in the view of coulomb and image force interaction with mask medium and particles.
	*Effects:* The best performance of filtration was observed in incidence of neutralized particles to the electrets filtration.

**Table 7:** The key elements of DBR machine are illustrated in Figure 16 [62].

Components	Role
Vacuum pump	simulates inhalation process
Compression pump	simulates exhalation process
Mass flow controller	monitors airflow rate with respect to certain breathing frequency
Virtual instrument	controls microelectronics system, mass flow controller and obtains the dynamic altering of airflow rate from mass flow controller and breathing resistance signals from microelectronics system
Microelectronic system	manages solenoid valve for changing the direction of air flow for the aim of exhalation and inhalation simulation
Pressure sensors	records dynamic changes of breathing resistance with regard to time

**Table 8:** The six indices presented for DBR measurement [62].

Indices	Unit	Diagram	Remarks
Maximum exhalation resistance (MER)	Pa	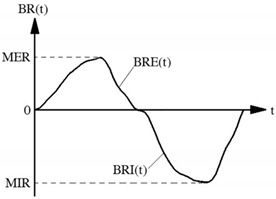	MER is defined as: ${\mathrm{MER}=\mathrm{BRE}(t)|}_{\max}$ in which BRE(t) shows breathing resistance with respect to time for exhalation process.
Maximum inhalation resistance (MIR)	Pa		MIR is defined as: ${\mathrm{MIR}=|\mathrm{BRE}(t)||}_{\max}$ in which BRI(t) shows breathing resistance with respect to time for inhalation process.
Average change rate of exhalation resistance (ACE)	Pa·S^−1^	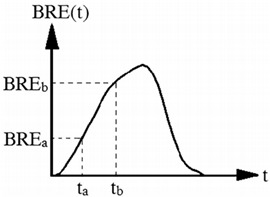	The slope of the exhalation resistance curve that is center 60% with regard to breathing resistance: $\mathrm{ACE}=\frac{{\mathrm{BRE}}_{b}-{\mathrm{BRE}}_{a}}{t_{b}-t_{b}}$ a and b refer to limits of the center 60% of the exhalation resistance curve according to BRE_a_ and BRE_b_, respectively.
Average change rate of inhalation resistance (ACI)	Pa·S^−1^	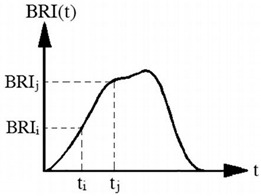	It is defined as center 60% of the slope of the inhalation resistance curve: $\mathrm{ACI}=\frac{{\mathrm{BRI}}_{j}-{\mathrm{BRI}}_{i}}{t_{j}-t_{i}}$ i and j refer to limits of the center 60% of the inhalation resistance curve according to BRI_i_ and BRI_j_, respectively.
Maximum change rate of exhalation resistance (MCE)	Pa·S^−1^	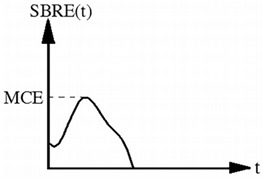	It is defined as the maximum slope of the exhalation resistance curve: ${\mathrm{MCE}=\mathrm{SBRE}(t)|}_{\max}$ SBRE(t) is the slope of exhalation resistance with regard to time.
Maximum change rate of inhalation resistance (MCI)	Pa·S^−1^	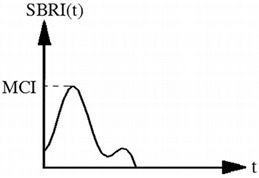	It is defined as the maximum slope of the inhalation resistance curve: ${\mathrm{MCI}=\mathrm{SBRI}(t)|}_{\max}$ SBRI(t) is the slope of inhalation resistance with regard to time.

## Data Availability

The data presented in this study are available on request from the corresponding author.
